# Comparison of patient and physician perspectives in the management of rheumatoid arthritis: results from global physician- and patient-based surveys

**DOI:** 10.1186/s12955-018-1035-3

**Published:** 2018-11-09

**Authors:** Allan Gibofsky, James Galloway, Joern Kekow, Cristiano Zerbini, Maria de la Vega, Gavin Lee, Eun Young Lee, Catalin Codreanu, Cheryl Koehn, Kathy Steinberg, Eustratios Bananis, Dario Ponce de Leon, Anna Maniccia, Ara Dikranian, for the RA NarRAtive global advisory panel

**Affiliations:** 1 Weill Cornell Medical College, 1300 York Avenue, New York, NY 10021 USA; 2 Hospital for Special Surgery, 535 East 70th Steet, New York, NY 10021 USA; 3 King’s College London and King’s College Hospital, Denmark Hill, London, SE5 9RS UK; 4 University of Magdeburg, Universitätspl. 2, 39106 Magdeburg, Germany; 5 Centro Paulista de Investigação Clinica, R. Moreira e Costa, 342 - Ipiranga, São Paulo, SP 04265-000 Brazil; 6 CEIM Investigaciones Médicas, Laprida 1307, Ciudad De Buenos Aires, 1425 Buenos Aires, Argentina; 7 Hong Kong Sanatorium & Hospital, 2 Village Road, Happy Valley, Hong Kong, SAR China; 8 Seoul National University College of Medicine, 103 Daehak-ro (Yeongeon-dong), Jongno-gu, Seoul, 03080 Korea; 9 Center for Rheumatic Diseases, University of Medicine and Pharmacy, 37 Dionise Lupu Street, 020021 Bucharest, Romania; 10 Arthritis Consumer Experts, 210–1529 West 6th Avenue, Vancouver, BC V6J 1R1 Canada; 11 Harris Poll, 155 Corporate Woods, Rochester, NY 14623 USA; 12 Pfizer Inc, 235 East 42nd Street, New York, NY 10017 USA; 13 Pfizer Inc, Calle Las Orquídeas 585, San Isidro, 15046 Lima, Peru; 14 Cabrillo Center for Rheumatic Disease, Suite 203, 300 S Pierce St, El Cajon, San Diego, CA 92020 USA

**Keywords:** Patient perspective, Rheumatoid arthritis, Treatment

## Abstract

**Background:**

In order to better understand the perspectives of patients and physicians regarding the treatment and management of rheumatoid arthritis (RA), we present and compare results from a patient-based and a physician-based survey developed by the RA NarRAtive advisory panel.

**Methods:**

The RA NarRAtive initiative is directed by a global advisory panel of 39 healthcare providers and patient organization leaders from 17 countries. A survey of patients self-reporting a diagnosis of RA and a physician-based survey, designed by the advisory panel, were fielded online by Harris Poll from September 2014 to April 2016, and from August 2015 to October 2015, respectively.

**Results:**

We present findings from 1805 patients whose RA was primarily managed by a rheumatologist, and 1736 physicians managing patients with RA. Results confirmed that RA carries a substantial disease burden; half of the patients surveyed reported stopping participation in certain activities as a result of their disease. While 90% of physicians were satisfied with their communications with their patients regarding RA treatment, 61% of patients felt uncomfortable raising concerns or fears with their physician. Of the patients providing responses, 52% felt that improved dialogue/discussion would optimize their RA management, and 68% of physicians wished that they and their patients talked more about their RA goals and treatment. Overall, 88% of physicians agreed that patients involved in making treatment decisions tend to be more satisfied with their treatment experience.

**Conclusion:**

The results of these surveys highlight the impact of RA on patients, and a discrepancy between patient and physician views on communication. Further research, focused on improving patient–physician dialogue, shared goal-setting, and treatment planning, is needed.

**Electronic supplementary material:**

The online version of this article (10.1186/s12955-018-1035-3) contains supplementary material, which is available to authorized users.

## Background

Rheumatoid arthritis (RA) is a chronic, progressive, disabling autoimmune disease with an estimated global prevalence of 0.24% [1]. Over time, a dysregulated immune-inflammatory process results in damage to the joints and other organs [2], causing pain, disability, and impaired health-related quality of life (HRQoL) [3]. RA is associated with a number of comorbidities, such as increased cardiovascular risk, which can result in higher rates of morbidity and mortality [4]. Consequently, optimum disease management is important to prevent disease progression and to improve long-term patient outcomes.

Current clinical guidelines define goals of RA treatment as remission, or low disease activity if remission is not possible [5, 6]. To meet these goals, guidelines recommend that patients with RA are initially treated with conventional synthetic disease-modifying antirheumatic drugs (csDMARDs). In patients who have an inadequate response or experience side effects to csDMARDs, escalation to more targeted therapies, either as monotherapy or in combination with csDMARDs, is recommended [5–7]. Although newer targeted therapies have greatly helped to improve the management of RA [8, 9], not all patients achieve remission, and many would like to change aspects of their treatment regimen [10, 11].

Due to the chronic nature of RA and the significant impact on HRQoL, the relationship between patients and their healthcare professionals is important for the implementation of appropriate treatment strategies [12, 13]. This is reflected in treat-to-target guidelines for the management of RA, which highlight the importance of shared decision making between patients and physicians [14]. Indeed, the first overarching principle in the current European League Against Rheumatism recommendations states that treatment of patients with RA “*must be based on a shared decision between the patient and rheumatologist”* [6], and the American College of Rheumatology recommend that “*treatment decisions should be made by physicians and patients through a shared decision-making process taking into account patients’ values, preferences, and comorbidities”* [5]. These decisions relate to all aspects of the disease, including risks, modalities of disease assessment, decisions on the therapeutic target, development of a management plan, and discussions on the benefits and risks of individual therapies. An understanding of both patient and physician perceptions of RA management could help foster more effective patient–physician relationships, which could lead to both increased patient satisfaction and improved treatment outcomes [15, 16].

Here, we present results from a patient-based and a physician-based survey developed by the RA NarRAtive global advisory panel. The inclusion of similar questions in both surveys allowed identification of similarities and differences in the perspectives of patients and physicians regarding RA treatment and management, while the geographic coverage of the surveys provided a global perspective.

## Methods

### Survey design and populations

The RA NarRAtive is an initiative sponsored by Pfizer Inc. and directed by a global advisory panel of 39 healthcare providers and patient organization leaders from 17 countries, with the aim of elevating the role of the patient in the management of RA [17, 18].

A patient-based survey was fielded by Harris Poll across 16 countries from September 2014 to April 2016. All patients were ≥ 18 years old and had self-reported that they had received a diagnosis of RA. In order to compare findings from the patient and physician surveys, results from the patient survey are only reported for respondents currently seeing a rheumatologist (or an orthopedist in Japan). Most patients were recruited from the Harris Poll Online Panel, a database of several million people who have agreed to participate in survey research; most patient surveys were fielded online (see Additional file 1).

A similar physician-based survey was fielded from August 2015 to October 2015. The physician questionnaire mirrored the patient questionnaire where applicable; questions were added based on research findings and feedback from the RA NarRAtive advisory panel. Physicians were asked to provide general information about all patients with RA for whom they were responsible, not individual patients. Therefore, no direct links could be established between responses collected in the patient and physician surveys. US physicians were recruited, via mail, from the American Medical Association Physician Master File. In all other countries, physicians were recruited from local online panels, using approaches that varied from country to country. All physicians reported seeing ≥5 patients in the previous month that they considered to have moderately to severely active RA.

The surveys were non-interventional and were not conducted as a clinical study; ethics approval was therefore not required.

Additional file 1 includes further details on the survey design, including some sample survey questions, together with additional information on the recruitment of the survey populations.

### Analyses of patient and physician surveys

Patient and physician responses were assessed using descriptive statistics, with standard t-tests to assess statistically significant differences at the 95% confidence level. Findings for patients from the USA were weighted by demographic variables (see Additional file 1). All other responses were not weighted. However, an adjustment was made to the global 16-country totals to account for the relative size of each country’s adult population within the total population of patients and physicians surveyed. Responses are included for 1805 patients primarily managed by a rheumatologist (or an orthopedist in Japan). All percentages are calculated based on a weighted base of 1764, and might not exactly match those derived by manual calculation due to weighting and/or computer rounding. Further details on analyses are provided in Additional file 1.

## Results

### Respondents

**Fig. 1:**
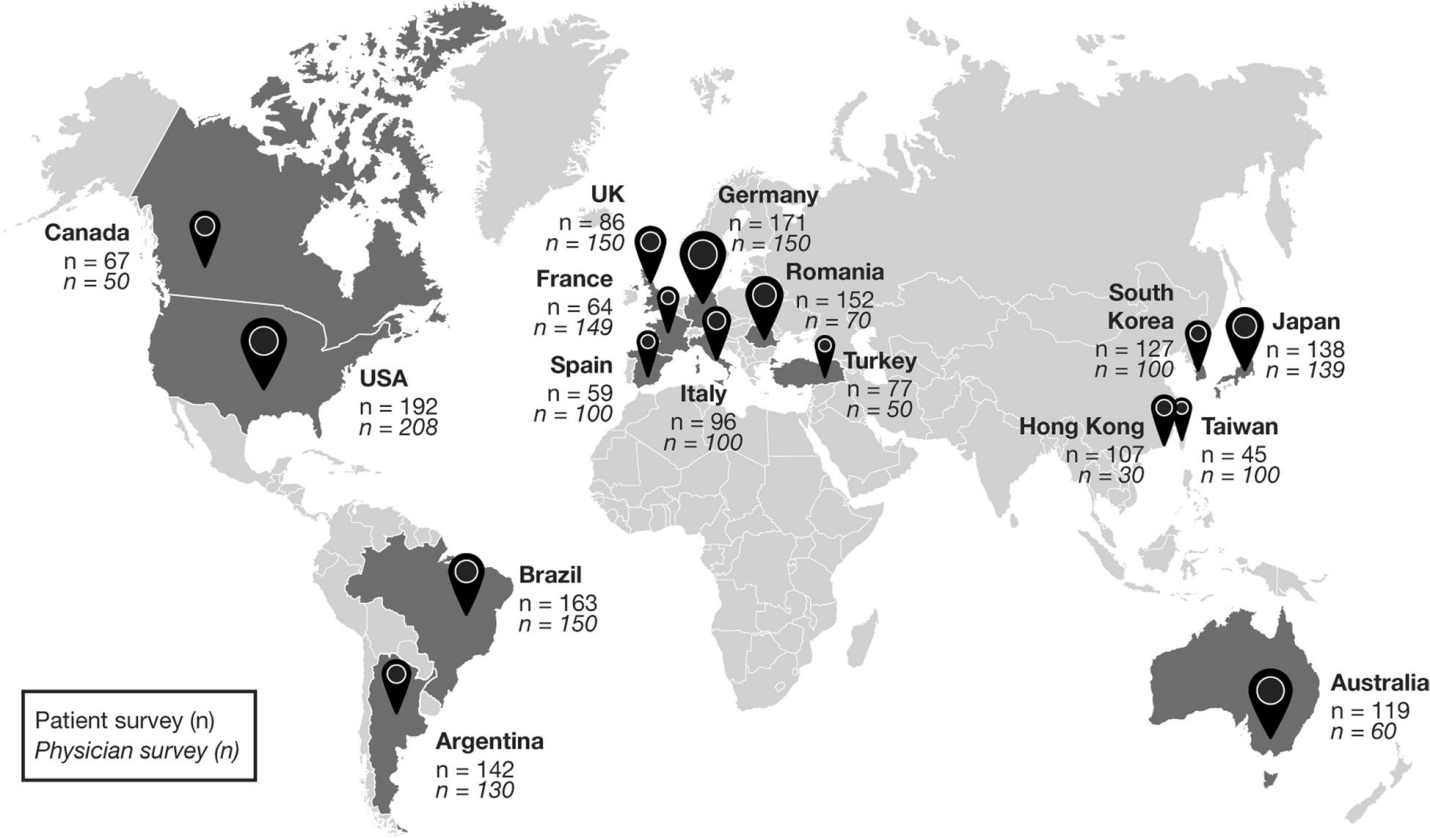
Global response to the RA NarRAtive patient and physician surveys. Abbreviation: RA, rheumatoid arthritis

Across 16 countries, 4170 patients with RA responded to the patient survey, of whom 1805 were primarily managed by a rheumatologist (or orthopedist in Japan) and were included in this analysis. A total of 1736 physicians responded to the physician survey (Fig. 1). Demographic details, as reported by patients, are given in Table 1. Overall, 67% of patients had been diagnosed with RA at least 5 years earlier, 33% self-reported moderate to severe disease activity, and only 31% described their current overall health as good/excellent (physicians were not asked to rate the overall health of their patients). Statistically significant differences were observed in the percentage of patients reporting good or excellent health between the USA (50%) and Taiwan, South Korea, and Spain (all < 10%) (*p* < 0.05).

**Table 1:** Patient demographics and disease characteristics

	Patient responders managed by a rheumatologist^a^ (*n* = 1805)^b^
Females, *n* (%)	1131 (64)
Mean age, years (SD)	51.7 (14.1)
Time since diagnosis, *n* (%)	
< 1 year	95 (5)
1–4 years	490 (28)
5–10 years	597 (34)
> 10 years	582 (33)
Median time since diagnosis, years (SD)	7 (9.8)
Current overall health, *n* (%)	
Good/excellent	550 (31)
Fair	892 (51)
Poor	322 (18)
Self-reported severity of RA, *n* (%)^c^	
Under control	610 (35)
Not under control	47 (3)
Mild	544 (31)
Moderate to severe	581 (33)
Severe	170 (10)

Abbreviations: *RA* rheumatoid arthritis, *SD* standard deviation

^a^In Japan, patients managed by an orthopedist were included

^b^1805 patients were included in the analysis, but not all patients provided responses to all questions; percentages were calculated based on the number of responses received

^c^Patients were permitted to select ≥1 response

In total, 51% of the physicians surveyed were office- or clinic-based and 22% were mostly hospital- or lab-based. Physicians saw a mean of 91 patients with RA per month. On average, physicians reported that 29% of their patients had mildly active disease, 33% had moderately to severely active disease, 17% had severely active disease, and 15% had uncontrolled disease.

### Impact of RA

Fifty-one percent of patients reported stopping participation in certain activities due to their disease. Thirty percent reported changing jobs (retiring from work completely, quitting a job, or switching jobs). Additionally, 5% had postponed having children (3% of male and 6% of female patients).

In total, 94% of patients had concerns relating to their RA. Patients most commonly worried about disease progression (75%), followed by the impact of the disease on HRQoL (72%). Treatment-related issues, including symptoms, treatment failure, exhaustion of treatment options, and flare-ups as a result of changed medication were causes for concern amongst 55% of patients, whereas 30% were worried about treatment access/cost. The relative importance of these topics was deemed similar by physicians when asked about the issues they believe their patients worry about, although treatment-related concerns and impact on HRQoL were both the second most commonly selected topics by physicians (88%). However, physicians were more likely to indicate that factors (e.g., worsening of RA, possible disability due to RA, RA will cause problems with job) were of concern to their patients, than patients were likely to indicate that concern.

### Patient–physician interactions

While 84% of patients were satisfied with the communication they have with their physician regarding their RA treatment, 55% indicated a desire to talk to their physician more about their RA treatment and goals. Furthermore, 52% felt that improved dialogue or discussion would help optimize their RA management. Patient satisfaction with their communication with their physician varied from country to country. For example, in the USA, Canada, Argentina, Brazil, and Romania, over 90% of patients strongly agreed/somewhat agreed that they were satisfied. Also, only 33% of patients from the USA, but 87% of patients from South Korea, wanted to talk to their physician more about their RA treatment and goals (*p* < 0.05).

In total, 61% of patients felt uncomfortable raising concerns or fears with their physician. When asked to provide reasons for this, 32% felt that their physician knows best and that they should follow what their physician tells them, and 31% worried that their physician would see them as a difficult patient and that it could affect the quality of care they receive. In addition, 21% felt that they did not have enough time to raise their concerns, or did not see their physician as often as they would like, and 14% felt they lacked knowledge or understanding. More patients who had been living with RA for < 5 years reported feeling uncomfortable raising their concerns compared with those who had been living with RA for 5 years or longer (69% vs. 57%, respectively; *p* < 0.05). Interestingly, patients who felt comfortable raising concerns and fears with their physician were more likely to describe their health as good/excellent than patients who felt uncomfortable (37% vs. 27%, respectively; *p* < 0.05).

Most (90%) physician respondents were satisfied with their communications with their patients regarding RA treatment. However, 68% wished that they and their patients talked more about their RA goals and treatment, and 66% wished that they could see their patients more often. The majority of physicians reported discussing HRQoL issues (93%), side effects/symptoms (93%), RA treatment (86%), and access/cost-related issues (63%) with their patients.

### Goals for RA management

**Fig. 2:**
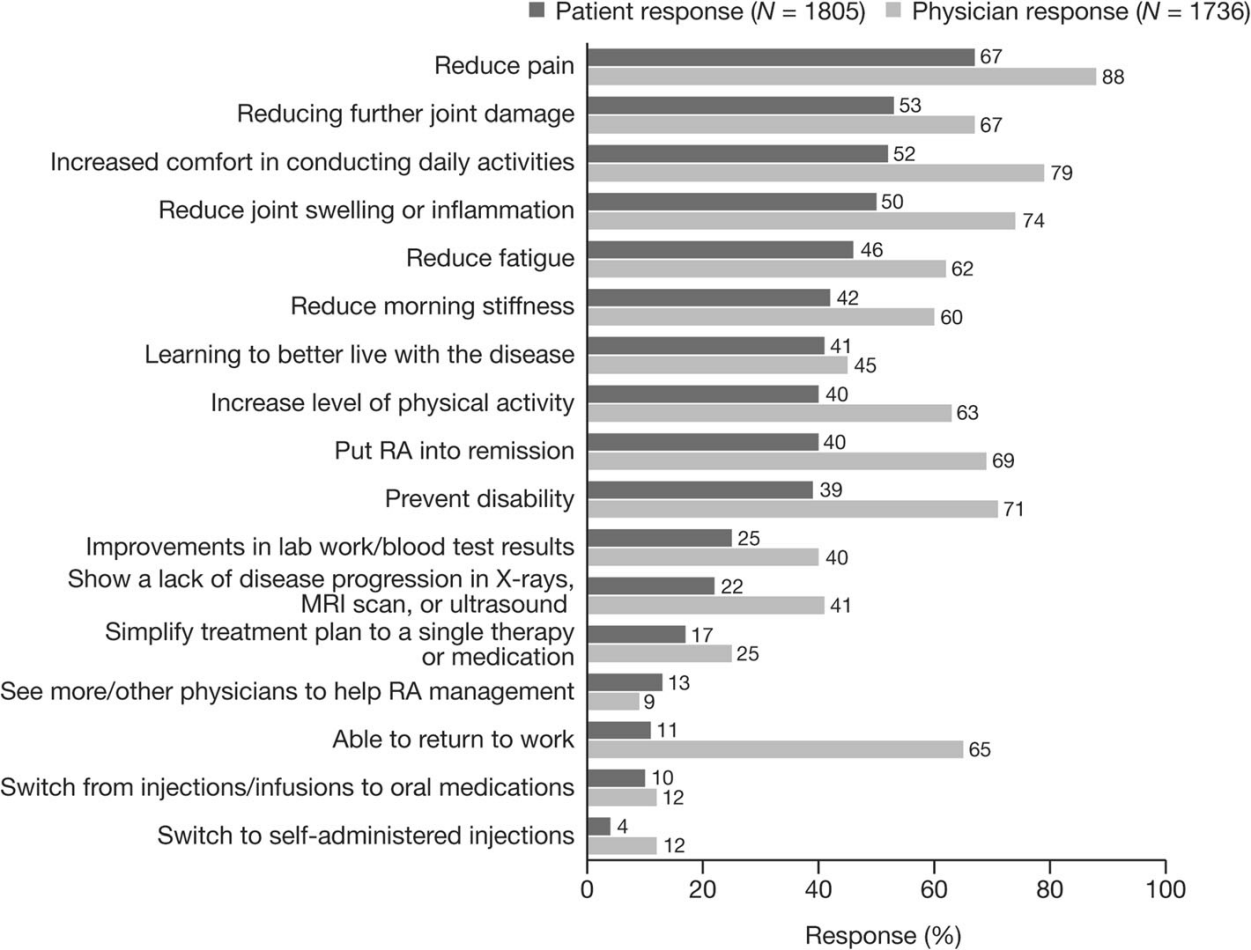
Goals reported by patients and by physicians for managing RA^a^. Abbreviations: MRI, magnetic resonance imaging; RA, rheumatoid arthritis. ^a^Patients were asked, “What are your goals for managing your RA?” and physicians were asked, “Based on what your patients tell you, what are your RA patients’ goals for managing their RA?”. Respondents were permitted to select ≥1 response

Nearly all patients surveyed (97%) had goals for managing their disease. The goals reported by patients for managing RA were similar to the goals reported by physicians based on their discussions with patients, with reduction in pain being the option selected by the highest proportion of both patients and physicians (Fig. 2). However, some goals were considered differently by patients and physicians. For example, reduction in further joint damage and fatigue were considered to be of higher relative importance in the patient survey than in the physician survey (Fig. 2). In contrast, preventing disability, RA remission, and ability to return to work were considered to be of higher relative importance in the physician survey than in the patient survey (Fig. 2). Only 44% of patients who had goals for managing their RA reported discussing their progress towards their treatment goals during every visit with their physician.

### RA treatment

**Fig. 3:**
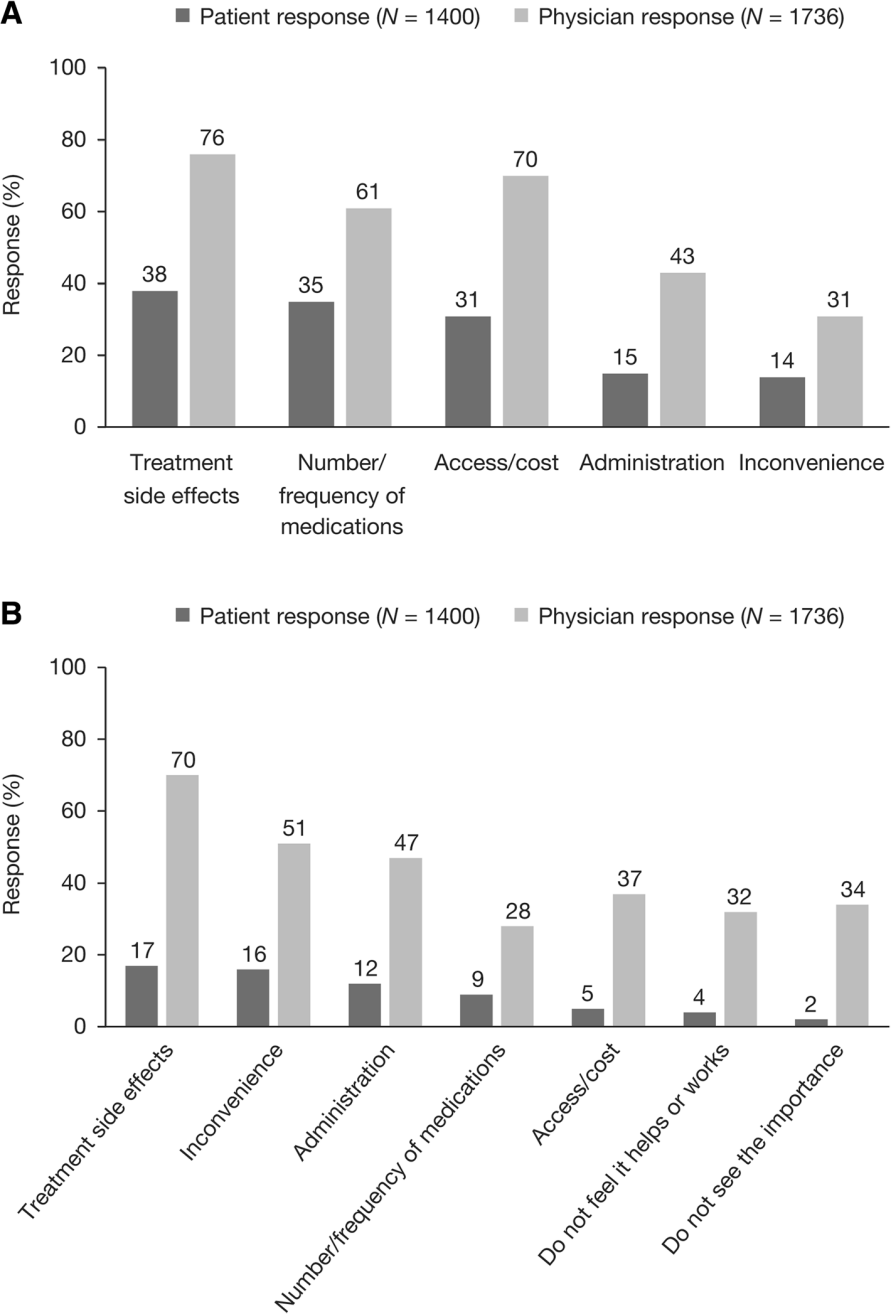
Aspects of RA medication that patients and physicians would most like to change (**a**)^a^, and most frequently cited reasons for non-adherence reported by patients and physicians (**b**)^b^. Abbreviation: RA, rheumatoid arthritis. ^a^Patients were asked, “Ideally, what would you most like to change, if anything, about your current RA medication(s)?” and physicians were asked, “Ideally, what would you most like to change, if anything, about currently available medications for RA?”. ^b^Patients were asked, “What are the most important reasons why you don’t take your RA medication(s) exactly as prescribed?” and physicians were asked, “Which of the following do you think are the top reasons why your patients don’t take their RA medication(s) exactly as prescribed?”. Respondents were permitted to select ≥1 response. Patient responses represent a base of patients currently taking prescription medication for their RA (*n* = 1400)

Overall, 76% of patients were taking prescription RA medication, which included pain relief (63%), csDMARDs (51%), corticosteroids (39%), and biologic DMARDs (24%). Most patients (81%) who were prescribed treatment for RA responded that they were satisfied with their treatment. However, 69% reported that they would ideally like to change something about their current RA medication. The most commonly reported aspects that patients would like to change were treatment side effects and the number/frequency of medications. Physician responses regarding the treatment aspects they would like to change for their patients were similar, although treatment access/cost was considered to be of higher relative importance by physicians (Fig. 3a).

In total, 36% of patients reported not taking their RA prescription medication exactly as prescribed. Rates of non-adherence were found to vary substantially from country to country, with some differences being statistically significant (*p* < 0.05); rates ranged from 15% in Argentina to 60% in Taiwan (with the highest adherence rate based on a very small number of patients). Globally, the most commonly cited reasons for non-adherence amongst patients included treatment side effects, inconvenience, and administration reasons (Fig. 3b). In the physician survey, similar reasons were cited (Fig. 3b).

Most (88%) physicians agreed that patients involved in making treatment decisions tend to be more satisfied with their treatment experience. Seventy-four percent felt that patients who are not involved are less likely to adhere to treatment. Furthermore, setting treatment goals with patients was considered absolutely essential/very important by 78% of physicians, and agreement on the treatment plan was considered to be absolutely essential/very important by 80% of physicians.

**Fig. 4:**
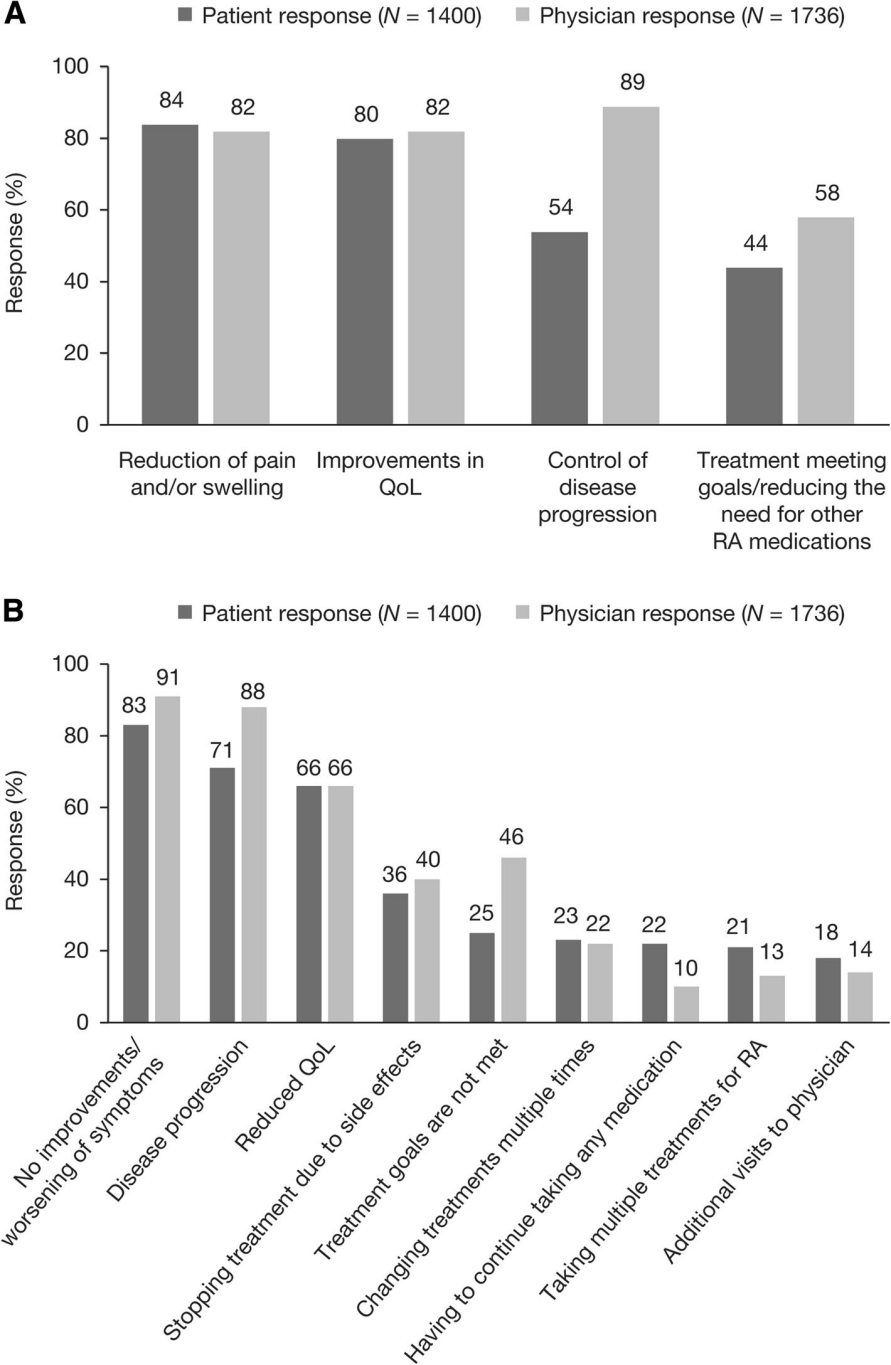
Criteria for defining treatment success (**a**)^a^ and treatment failure (**b**) ^b^, as selected by patients currently receiving prescribed treatment for RA and by physicians. Abbreviations: RA, rheumatoid arthritis; QoL, quality of life. ^a^Patients were asked, “When thinking about your RA medication(s), what does ‘successful’ treatment mean to you?” and physicians were asked, “When thinking about the RA medications that are currently available, what does “successful” treatment mean to you?” ^b^Patients were asked, “When thinking about your RA medication(s), what does ‘failure’ mean to you?” and physicians were asked, “When thinking about the RA medications that are currently available, what does ‘failure’ mean to you?”. Respondents were permitted to select ≥1 response. Patient responses represent a base of patients currently taking prescription medication for their RA (*n* = 1400)

Successful treatment was most commonly defined by patients as a reduction in pain and/or swelling, whereas physicians most commonly defined treatment success as the control of disease progression (Fig. 4a). The definitions provided by patients and physicians for treatment failures were similar and included no improvements/worsening of symptoms, disease progression, and reduced HRQoL (Fig. 4b).

### Future management of RA

**Fig. 5:**
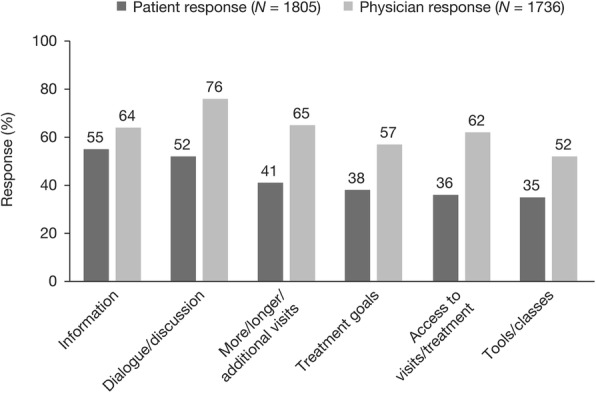
Factors that patients and physicians feel would help them to more successfully manage RA^a^. Abbreviation: RA, rheumatoid arthritis. ^a^Patients were asked, “In thinking about your relationship with your doctor or healthcare professional, which of the following, if any, would help you more successfully manage your RA?”. Physicians were asked, “In thinking about your relationship with your moderate to severe or severe RA patients, which of the following, if any, would help you more successfully manage your patients’ RA?”. Respondents were permitted to select ≥1 response

Patients most commonly reported that information and dialogue/discussion are aspects of their interactions with their physicians that would help them to manage their RA more successfully (Fig. 5). Amongst physicians, discussion/dialogue was the most commonly reported topic for the successful management of RA, followed by more/longer/additional visits.

## Discussion

In this paper, we present results from two related global surveys: one patient-based, and one physician-based. The surveys were developed by the RA NarRAtive global advisory panel to better understand the perspectives of both patients and physicians with regards to RA management. We explored themes relating to the broader impact of RA, patient–physician interactions, RA treatment goals, satisfaction with treatment, and RA management. Questions in the physician survey mirrored those in the patient survey, where appropriate. Although the survey populations were independent of each other, with no direct link between the responses from patients and those from physicians, this approach allowed identification of differences in the perspectives of patients with RA and physicians who manage and treat patients with RA. The fielding of the survey in 16 countries also provided a global perspective, allowing identification of differences in the views and attitudes of patients and physicians between countries.

A large proportion of patients reported stopping participation in certain activities (51%) or changing jobs (30%) as a result of their disease. The finding related to changing jobs is extremely interesting. It suggests that the introduction of more targeted therapies has not reduced the impact of RA on work that was reported before biologics were widely available [19, 20], although it is important to remember that only 24% of patients included in this survey were receiving targeted treatment. Furthermore, many patients worried about the impact of their RA on HRQoL. It is well known that RA can have a significant impact on patients’ HRQoL [3, 21]. Consistent with this, the results presented here suggest that some patients may not be receiving optimal disease management, despite the availability of numerous effective treatments and treatment regimens.

Most physicians (90%) were satisfied with their interactions with patients, while 84% of patients were satisfied with communication regarding RA treatment. This slight disparity is interesting and might reflect a lack of insight on the part of a small proportion of physicians regarding their relationship with their patients. Some physicians wished that treatment and goals could be discussed in more depth during clinical visits with their patients, and many wished they could see their patients more often. Similarly, some patients indicated a desire to talk to their physician more about their RA goals and treatment; the proportion of patients varied markedly from country to country, with 87% of patients from South Korea, but only 33% of those from the USA, selecting this option. Over half of the patients responding acknowledged that improved dialogue or discussion would help to optimize their RA management. The surveys also showed that patients who described their current overall health as good/excellent were more likely to feel comfortable raising their concerns or fears with their physician than those whose health is not as good. This might reflect a positive relationship between patients and physicians, together with a level of confidence and trust in the physician, when patients are in good health. Taken together, these findings highlight the importance of effective patient–physician dialogue in the management of RA, and suggest that patients’ perceptions of their relationships with their physician can positively impact the management of their disease. This is in line with previous reports suggesting that effective patient–physician dialogue is important for optimum RA management [12, 13].

Although newer therapies have enabled physicians to improve the management of RA, non-adherence persists, and many patients would like to change aspects of their treatment. Non-adherence rates varied substantially by country, possibly reflecting social and cultural differences in the patient–physician relationship between countries, and highlighting the risks of extrapolating findings in one country or region to others. Treatment adherence in patients with RA is known to be low, and has been shown to vary from 30% up to 80% [22].

Increasing the length of the consultation [23], and greater involvement with social support groups [24], could help to improve adherence and, consequently, treatment efficacy. While a large proportion (81%) of patients surveyed were satisfied with their RA treatment, 33% considered their RA to be moderate to severe, and only 35% described their RA as under control. This suggests a disconnect between self-reported treatment satisfaction and self-described status of disease that might impact RA management. Also, despite the high proportion of patients reporting that they were satisfied with their treatment, 69% would ideally like to change something about their current RA medication. Given the need to limit respondent burden, detailed questions on the link between treatment satisfaction and desire for change were not included in the survey. However, as side effects and the number/frequency of medications were the treatment aspects that patients would most like to change, this might reflect a desire for these aspects to be improved.

While there were some similarities in the results of the patient and physician surveys, the relative importance assigned to issues frequently varied between the two respondent populations. For example, differences in the goals reported by physicians and patients were identified, as were differences in the definitions of treatment success and treatment failure. Some differences were considerable: 65% of physicians selected being able to return to work from the list of goals for managing RA, while only 11% of patients selected this option; respondents could choose multiple options from the list. Additionally, while 89% of physicians indicated the control of disease progression as indicative of successful treatment, only 54% of patients selected this option, and 46% of physicians selected not meeting treatment goals as a definition of treatment failure, compared with only 25% of patients.

One challenge may be that terms such as ‘remission’ may mean something different to physicians compared with patients, and maybe even between different physicians. As in everyday interactions, semantics plays a role during an open dialogue and discussion. Therefore, establishing a common meaning for certain words is of paramount importance. Acknowledging these differences and challenges could lead to improved patient satisfaction and treatment adherence. This is consistent with the feeling of most physicians in the current survey that patients who are involved in making treatment decisions tend to be more satisfied with their treatment experience, and those who are not involved are less likely to adhere to treatment. Differences were also identified in the topics that patients worry about, and the topics that physicians believe their patients worry about. Increasing physician awareness of patients’ fears and concerns may encourage physicians to probe in more depth when assessing the disease activity status of their patients and setting treatment goals.

Shared decision making between physicians and patients when establishing treatment goals is widely acknowledged as best practice [5, 15], Furthermore, the importance of considering the patient’s perspective regarding treatment is emphasized by the Outcome Measures in Rheumatology (OMERACT) international consensus initiative [25]. In line with these and other recent reports, these surveys emphasize the importance of taking into account patient needs with respect to treatment decisions in order to improve patient satisfaction and treatment outcomes [26, 27].

While to our knowledge this is the first report relating to RA that incorporates the perspectives of both patients and physicians via the same or similar survey questions, some of the themes identified are similar to those reported previously. For example, in a survey of patients from the Netherlands, the United Kingdom, Austria, Denmark, France, and the USA, patients reported that they would like to improve pain, fatigue, independence, mobility, and physical functioning [28]. Our results are also consistent with those reported in the Rheumatoid Arthritis: Insights, Strategies & Expectations (RAISE) survey, which reported a substantial impact of RA on HRQoL. As was found here, only a minority of the patients in the RAISE survey discussed their issues with their physician [29]. In a survey of physicians, the level of agreement with 10 international recommendations for treating RA was measured, and the highest ranking statement was ‘the primary target for treatment of RA should be a state of clinical remission’ [30], which was also the highest ranking option for successful treatment selected by physicians in the survey described here. Further understanding of the responses from these surveys will be important to encourage the facilitation of continued communication between patients and physicians with the aim of improving outcomes.

As with any patient-based survey, the interpretation of the patient survey findings were limited by the survey being strictly self-reported by patients with RA, therefore relying on accurate patient recall of disease management and their understanding of questions and their diagnosis of RA. Another possible limitation was the method for inclusion of respondents. The approach involved including a convenience sample of both patients and physicians, which introduced the potential for selection bias. This was predominantly an online survey, hence patients were likely more computer literate and active online than the general population and, therefore, may not be representative of all patients with RA. While a weighted target approach was used for patients from the USA in order to balance the sample for patient demographics, for other countries where weighting targets were unavailable, the results are only representative of the individuals sampled and may not be representative of the general population. A relatively high proportion of patients who completed the survey reported that they were not receiving treatment with DMARDs; this could influence outcomes, particularly if patients were not aware that their medication is a DMARD. In countries where some or all patients were recruited from local patient organizations (South Korea, Taiwan, Hong Kong), there may have been additional or different types of selection bias (e.g., membership of the patient organization could indicate patients more engaged in understanding their disease and improving outcomes than average). Also, in Argentina, where a portion of the interviews were conducted face to face, interviewer bias and/or social desirability bias may have influenced both willingness to participate and respondents’ answers to some questions (e.g., under-reporting of non-adherence). The physician survey also relied on accurate recall and reporting by physicians, as well as internet access. Furthermore, although the surveys were fielded in 16 countries, findings may not be applicable to all countries/regions due to regional differences in the management of RA. Cultural and economic differences, as well as differences in healthcare systems and access to RA treatments between countries, may have influenced participant responses to this survey. Geographic differences were observed in several parameters, including patients’ satisfaction with their communication with their physician and treatment adherence; these might be even greater compared with some countries not included in this survey. The authors acknowledge that this survey did not explore all recognized comorbidities and risk factors associated with RA, such as cardiovascular disease. This reflects the need to keep the survey to a manageable length, and the focus on communication and dialogue between physicians and patients, in order to determine the most important issues for both groups and ultimately improve the management of RA.

## Conclusion

In conclusion, although patients and physicians placed similar importance on communication, the survey results highlighted important disconnects between patients and physicians, e.g., in regard to the importance of returning to work as a treatment goal. Geographic differences were also apparent, with large variations in the level of medication adherence reported by patients from different countries. The findings suggest that, at least in a proportion of cases, improved patient-physician communication could lead to improved management of RA. Almost two-thirds of patients in this survey felt uncomfortable raising concerns or fears with their physician. Improved dialogue between physicians and patients may provide the opportunity for patients to more openly express any expectations or concerns, allowing physicians to take actions to meet the specific requirements of individual patients. Further research on the reasons for the observed discrepancies between the views of patients and physicians, and across the various countries, could help inform efforts to improve interactions between patients with RA and those managing their healthcare, and provide guidance or tools to optimize communication between patients and physicians within limited consultation times. Overall, greater understanding and awareness of discrepancies in patient and physician experiences could drive better communication and patient engagement, and possibly lead to improved adherence and overall patient satisfaction. The RA NarRAtive initiative is continuing to focus on developing tools and resources that can bridge these identified gaps to improve patient–physician dialogue and help improve overall management of RA.

## Additional file

Additional file 1 Appendix. Further details on the survey design, including some sample survey questions, together with additional information on the recruitment of the survey populations. (DOCX 26 kb)

## Abbreviations

csDMARDs: Conventional synthetic disease-modifying antirheumatic drugs
DMARDs: Disease-modifying antirheumatic drugs
HRQoL: health-related quality of life
OMERACT: Outcome Measures in Rheumatology
RA: Rheumatoid arthritis
RAISE: Rheumatoid Arthritis: Insights, Strategies & Expectations
SD: Standard deviation
