# Supplementary material for: Comparison of patient and physician perspectives in the management of rheumatoid arthritis: results from global physician- and patient-based surveys
Source: Health Qual Life Outcomes. 2018 Nov 9;16:211. doi: 10.1186/s12955-018-1035-3 (PMC6230272; doi:10.1186/s12955-018-1035-3)
Supplement: Supplementary file 1 — Appendix. Further details on the survey design, including some sample survey questions, together with additional information on the recruitment of the survey populations. (DOCX 26 kb) [file 12955_2018_1035_MOESM1_ESM.docx]

**Additional file 1: Appendix**

**Methods**

**Survey design**

Questions were initially based on rheumatoid arthritis (RA) signs and symptoms described in many textbooks and articles regarding clinical practice. To further inform survey development, an audit of ‘white space’ in research on RA was conducted, following which the survey instrument was designed with input obtained internally and from the RA NarRAtive advisory panel of patients and physicians, including rheumatologists in everyday clinical practice. A pilot test was conducted among patients in the USA and resulted in minimal edits to the survey patient questionnaire.

Questions on both the patient and healthcare professional (HCP) questionnaires required respondents to provide a numeric response, to select a single option or multiple options from a list, or to indicate their level of agreement with a statement (from ‘*Strongly disagree’* to ‘*Strongly agree*’).

A number of questions were included that addressed related issues. Two examples follow:

(1) Patients were asked:
“*With respect to your communication with the doctor or healthcare professional who is mostly responsible for managing your RA, how much do you agree or disagree with each of the following statements?*”
Two of the statements were:
“*I am satisfied with the communication I have with my doctor or healthcare professional about my RA treatment*”
and
“*I wish my doctor or healthcare professional and I talked more about my RA goals and treatment*”.

(2) HCPs were asked:
*“About how many RA patients have you seen in the past month whom you would categorise as…*?” and were asked to provide the number of patients in each of the categories: mildly active, moderately to severely active, severely active, or in remission.

In a separate question, HCPs were also asked, “*What proportion of all your RA patients have RA that is uncontrollable?”.*

Findings were analyzed as reported by the respondents, with no reconciliation of apparently contradictory responses to related questions.

***Recruitment of survey populations***

A proportion of surveys in Taiwan (40/90) and Hong Kong (79/192), and all those in Romania (183/183), were conducted via a paper survey that was distributed by local patient organizations. In Romania, the Romanian League against Rheumatism helped with the logistics of the survey, but not with patient selection. In South Korea, 56/224 surveys were conducted utilizing a public link that was distributed by a local patient organization via email and Facebook. In Argentina, responses for most patients (162/217) were collected via face-to-face interviews with patients recruited from local health centers. All other patients were recruited (and completed the survey) online, with samples sourced from the Harris Poll Online Panel and other online panel partners as needed. Due to the various ways in which respondents were recruited across countries, neither the total number of respondents recruited or the actual response rate achieved could be calculated.

The physician-based survey was also fielded by Harris Poll. Questions were added to this survey based on the ‘white space’ identified in the audit of research on RA, as well as feedback/questions received from the RA NarRAtive advisory panel during presentation of the patient survey results.

In Romania, all physicians, and in Japan, all orthopedists, were required to prescribe biologics in order to be included.

**Analyses of patient and physician surveys**

The online survey prevented respondents proceeding to the next question without providing an answer to the current question; items without a response in the paper-based survey were coded as ‘Respondent left blank’. Findings related to RA treatment were based on patients currently taking prescription RA medication, rather than the total patient population.

Demographic variables used to weight findings for patients from the USA included age within gender, education, race/ethnicity, region, income, marital status, and internet usage. At the time the research was conducted, weighted targets were only available for patients from the USA.
